# Physicochemical, Nutritional, and Antioxidant Properties of Traditionally Fermented Thai Vegetables: A Promising Functional Plant-Based Food

**DOI:** 10.3390/foods13172848

**Published:** 2024-09-08

**Authors:** Wanida Pan-utai, Sarn Settachaimongkon, Orawan La-ongkham, Soisuda Pornpukdeewattana, Marisa Hamwane, Chalantorn Lorpeunge, Masnavee Adame, Charisa Yodbumprenge

**Affiliations:** 1 Department of Applied Microbiology, Institute of Food Research and Product Development, Kasetsart University, Bangkok 10900, Thailand; ifrowl@ku.ac.th; 2 Department of Food Technology, Faculty of Science, Chulalongkorn University, Bangkok 10330, Thailand; sarn.s@chula.ac.th; 3 School of Food Industry, King Mongkut’s Institute of Technology Ladkrabang, Bangkok 10520, Thailand; soisuda.po@kmitl.ac.th (S.P.); marisahamwan@gmail.com (M.H.); chalantornlp@gmail.com (C.L.); masnavee.ad@gmail.com (M.A.); charisa.yb@gmail.com (C.Y.)

**Keywords:** indigenous food, fermentation, diversity, functionality, authenticity

## Abstract

Fermented plant-based products were gathered from various regions in Thailand and categorized into 10 types of traditional commercial vegetables. Different vegetable materials and natural fermentation methods influence the diverse physical, chemical, nutritional, and functional attributes of the products. All the traditionally fermented Thai vegetable samples collected showed physicochemical properties associated with the fermentation process, contributing to the nutritional and functional quality of the final products. Achieving consistent research results is challenging due to the intricate nature of food matrices and biochemical processes during fermentation. The roles of microorganisms, especially probiotics, are crucial in delivering health benefits through fermented foods. Traditionally fermented Thai vegetable foods contain high levels of total soluble solids, titratable acidity, and salinity in pickled shallot and ginger as a result of the natural fermentation process and the ingredients used. The research findings were confirmed using a hierarchical cluster analysis (HCA)-derived dendrogram pattern. The nutritional compositions, total phenolic contents, and antioxidant activities varied among the different types of vegetables. The correlations among lipid, protein, fiber, total soluble solid (TSSs), total titratable acidity (TTA), and salinity as potential biomarkers in fermented vegetable products were examined. The results suggest that traditionally fermented Thai vegetable products significantly impacted food research by enhancing the quality and preserving the authenticity of traditionally fermented Thai vegetables.

## 1. Introduction

The current food landscape is characterized by a growing emphasis on healthy, sustainable, and plant-based dietary practices. This has led to heightened consumer interest in diversifying their vegetable intake [1]. Vegetables are widely acknowledged for their nutritional benefits, providing essential vitamins, minerals, and dietary fibers that play a crucial role in supporting overall health and well-being [2]. The rising importance of vegetables in ensuring food and nutrition security underscores their significance in promoting long-term food sustainability and accessibility [3].

Fermented foods have played a pivotal role in human diets since ancient times, with early evidence in Asia dating back to around 8000 B.C. [4]. Nowadays, fermented foods are produced through controlled microbial growth and enzymatic action, comprising 5% to 40% of all globally consumed food [5]. The beneficial effects of fermented foods on health have cemented their significance in human diets. Active microorganisms enhance the availability of nutrients and promote probiotic and prebiotic functions, ultimately improving food nutritional properties and health benefits [6]. These microorganisms must remain viable during digestion to carry out their functions in the intestinal tract including modifying the gut microbiota and facilitating fermentation [7]. Fermentation is a traditional method predating refrigeration that has long been used to prolong the shelf life of perishable foods [8]. This process involves the conversion of sugars and other carbohydrates into alcohols or organic acids using microorganisms such as yeast, bacteria, or fungi. Fermentation inhibits the growth of harmful bacteria and preserves the food by creating an acidic or alcoholic environment [9]. This method was crucial in ancient times when refrigeration was unavailable, allowing people to store and consume food over extended periods [10]. Fermentation is widely used to create various foods and beverages with improved flavor, texture, and shelf life [11]. Fermented vegetables contain lactic acid bacteria, which exhibit probiotic characteristics including the reduction and assimilation of cholesterol, antioxidant activity in vitro and in vivo, anticancer activity, immunomodulatory activity in vitro, and inhibitory activity against Gram-negative and Gram-positive pathogenic bacteria responsible for prevalent foodborne diseases worldwide [12,13]. 

A previous survey revealed that the hill tribes in Northern Thailand utilize over 1000 plant species. The considerable plant diversity observed in the fresh food markets provides insight into Thailand’s rich bio-cultural diversity [14]. Previous research on traditionally fermented Thai food products reported the benefits of bacterial communication, as a rich source of functional and probiotic foods [15]. Traditionally fermented foods result from spontaneous fermentation processes. Fermented foods in Asian cultures undergo fermentation utilizing lactic acid bacteria (LAB) [16]. The intricate nutritional composition of raw food materials and vegetables serves as a bountiful source of essential vitamins and minerals conducive to the proliferation of LAB strains, thereby facilitating the microbial synthesis of enzymes and other metabolites [17]. Fermentation has been used for generations in Southeast Asia to create food, with several food products in the region gaining global renown [18]. Even with advancements in agriculture, fermented products in Southeast Asia are typically still crafted using traditional methods on a small scale, often within families or villages [19]. Fermented fruits and vegetables play a crucial role in providing food for people on all continents [11]. They are essential for preserving and producing nutritious foods with a wide range of flavors, aromas, and textures that enhance the human diet and eliminate antinutritional factors to ensure food safety [20]. Fermentation offers numerous benefits such as improving food security, enhancing nutrition, and contributing to the social well-being of marginalized and vulnerable communities [21]. Fermentation-based industries provide a significant source of income and employment opportunities in Asia [11,22]. 

Functional foods play a crucial role in preventing and managing non-communicable diseases (NCDs) and have been extensively researched [23]. Fermentation involves the action of microorganisms to transform the food and enhance its digestibility and nutritional value by releasing bioactive compounds [24]. Bioactivity improves during fermentation due to the release of trapped compounds, the production of metabolites, and the metabolic products of microorganisms [25]. Fermented foods contribute beneficial live microbes to the gut, promoting gut health [26]. Most studies demonstrated increased bioactivity during fermentation, with conflicting results attributed to the complexity of the food, types of microbes used, and environmental conditions during fermentation [27,28]. Vegetables possess various nutritional and functional properties, but limited information is available on the physicochemical, nutritional, and functional properties of traditionally fermented Thai vegetable products. 

The main goal of this research was to explore fermented products made from traditional Thai vegetables. The physicochemical, nutritional, and functional properties of these products were analyzed to assess their potential for developing functional plant-based foods.

## 2. Materials and Methods

### 2.1. Fermented Vegetables

Ten traditionally fermented Thai vegetable products comprising 45 samples were collected from local markets in the northern, southern, western, eastern, northeastern, and central regions of Thailand, as detailed in Table 1. These products were pickled pak-kum, pickled pak-sian, pickled bamboo shoots, pickled stink beans, pickled shallot, pickled mustard greens, pickled ginger, fermented tea leaves, pickled scallions, and pickled bud rieng. 

### 2.2. Physicochemical Properties

The traditional Thai food product samples consisted of two main parts: vegetable solids and fermentation broth. Before physicochemical and functional analyses, the vegetable solids and fermentation broth were mixed in equal proportions using a Stomacher (BagMixer^®^ 400 CC, Interscience, Paris, France). The total soluble solid (TSS) content was quantified using a digital refractometer (PAL-1, Atago, Tokyo, Japan). A pH meter (Lab850, Schott Instruments GmbH, Mainz, Germany) was used to determine the pH levels. The total titratable acidity (TTA) percentage was determined using 0.1 N sodium hydroxide solution (KenAus™, Cherrybrook, New South Wales, Australia) with phenolphthalein as an indicator [29]. The percentage of salinity as sodium chloride content was measured using a digital refractometer (HI96821, HANNA, Sălaj, Romania) according to the manufacturer’s instructions. 

### 2.3. Nutritional Composition

The fermented sample products were oven-dried (Universal Oven, Memmert, Schwabach, Germany) at 55–60 °C for 20–24 h to reduce the moisture content to 10% (*w*/*w*) and then milled to 0.5–1 mm particle size. The chemical compositions of the fermented vegetable products were analyzed according to AOAC standard methods [30]. Briefly, the Kjeldahl method was used to determine crude protein content, with crude lipid obtained through Soxhlet extraction using petroleum ether. The resulting lipid was dried until constant weight. Both acid and alkaline digestion methods were utilized to assess crude fiber content, and the fiber residue was also dried until constant weight. The ash content was determined by igniting the dried samples in an electric furnace at 550 °C, while the carbohydrate content was calculated by subtracting the sum of moisture, protein, lipid, fiber, and ash contents from 100 g of dry matter.

### 2.4. Total Phenolic Content

The total phenolic content (TPC) was determined using the Folin–Ciocalteu method, as outlined by Pan-utai et al. (2023) [31]. In summary, 20 µL of the sample or standard was combined with 100 µL of 10% Folin–Ciocalteu reagent in a 96-well plate, and the mixture was incubated in the dark at room temperature for 8 min. Then, 80 µL of 7.5% sodium carbonate and 50 µL of DI water were added and mixed thoroughly before incubating at 40 °C for 30 min. The absorbance was measured at 750 nm using a microplate reader (M965+, Microplate Reader, Metertech, Taipei, Taiwan) with gallic acid as the standard, and TPC was expressed as milligrams of gallic acid equivalent per gram of dried biomass (mg GAE/g).

### 2.5. DPPH Radical Scavenging Activity

The DPPH radical scavenging activity assay was performed following Hung et al. [32] with minor modifications. In summary, a 100 µL sample or standard was combined with 100 µL of 200 µM DPPH solution (2,2-diphenyl-1-picrylhydrazyl, Sigma-Aldrich, Queenstown, Singapore). After a 30 min incubation in a light-free environment at room temperature, the absorbance was measured at 517 nm using a microplate reader (M965+, Microplate Reader, Metertech, Taiwan). The percentage of inhibition was calculated using the following equation:

$$\mathrm{Inhibition}\;\left(\%\right)=\frac{OD_{control}-OD_{sample}}{OD_{control}}\times 100$$

### 2.6. ABTS Radical Scavenging Activity

The ABTS radical scavenging activity was carried out following the procedure outlined by Pan-utai et al. (2023) [31]. Briefly, 505.05 µL of 245 mM of ammonium persulfate was mixed with 5.05 µL of 7 mM of ABTS. The mixture was kept in the dark at room temperature for 16 h and then diluted with deionized water to achieve an absorbance of 0.7 at 750 nm. Then, 190 µL of the ABTS solution was combined with either the sample or the standard in 10 µL amounts in a 96-well plate and incubated for 5 min at room temperature in the dark. The absorbance was measured at 750 nm using a microplate reader (M965+, Microplate Reader, Metertech, Taipei, Taiwan). The percentage of inhibition was calculated by the following equation:

$$\mathrm{Inhibition}\;\left(\%\right)=\frac{OD_{control}-OD_{sample}}{OD_{control}}\times 100$$

### 2.7. Statistical Analysis

The results were presented as mean values with standard deviation (SD) for each triplicate experiment. Statistical analysis was performed using SPSS (SPSS, Inc., Version 25.0, Armonk, NY, USA). All experiment parameters were compared using Duncan’s multiple range test (DMRT) at a significance level of 0.05.

To compare the overall nutritional profiles among the samples, the values from all parameters were normalized and subjected to multivariate analysis using the MetaboAnalyst 6.0 online platform (www.metaboanalyst.ca, accessed on 13 July 2024). Heatmap visualization combined with Pearson’s correlation-based hierarchical cluster analysis (HCA) and partial least squares discriminant analysis (PLS-DA) were applied to evaluate the distinctive patterns of sample chemical profiles. Chemical parameters with variable importance in projection (VIP) scores > 1.0 and *p* ≤ 0.05 were considered potential components responsible for the distinction of samples. Pearson’s correlation analyses among the chemical parameters were performed, and the results were depicted as hierarchically clustered correlation matrices and correlation pattern plots. 

## 3. Results

**Figure 1:**
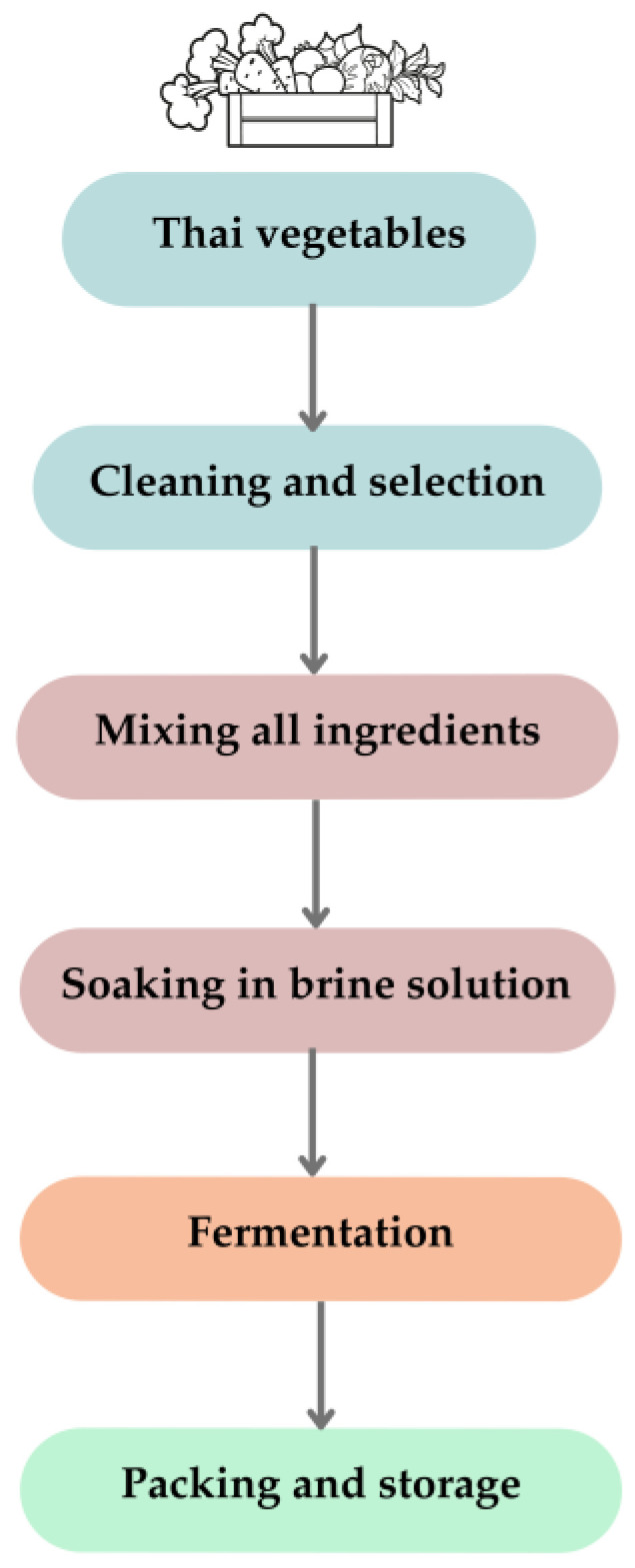
The overall fermentation process of traditional Thai vegetables. Adaptation from Swain, Anandharaj, Ray, and Parveen Rani [11] and Yuan et al. [33].

**Figure 2:**
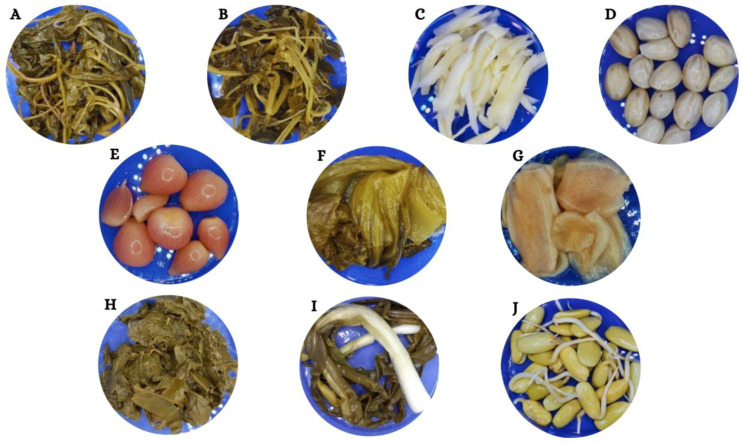
Physical appearance of traditionally fermented Thai vegetables. (**A**) Pickled pak-kum, (**B**) Pickled pak-sian, (**C**) Pickled bamboo shoots, (**D**) Pickled stink beans, (**E**) Pickled shallot, (**F**) Pickled mustard greens, (**G**) Pickled ginger, (**H**) Fermented tea leaves, (**I**) Pickled scallions, and (**J**) Pickled bud rieng.

Ten popular types of fermented vegetables were obtained. These ten traditionally fermented Thai vegetables are popular throughout Thailand and distributed to consumers nationwide. The overall fermentation processes of traditional Thai vegetables are shown in Table 2 and Figure 1. The physical appearances of traditionally fermented Thai vegetable products collected from various regions and locations in Thailand are shown in Figure 2. The fermented vegetable products were made using different plant parts. The samples were categorized as leaves, tubers, and seeds or beans. Fermented vegetables from the leaves included pickled pak-kum (A), pickled pak-sian (B), pickled mustard greens (F), fermented tea leaves (H), and pickled scallions (I). By contrast, the tuber part of the vegetables comprised pickled bamboo shoots (C), pickled shallot (E), pickled ginger (G), and pickled stink beans (D). Pickled bud rieng (J) was obtained from the seeds. A wide variety of traditional Thai vegetables displayed unique characteristics and contributed diverse qualities to plant-based products through the fermentation process. This study examined the physicochemical, nutritional, and functional properties of fermented products made from traditional Thai vegetables and compared the correlations among these properties.

### 3.1. Physicochemical Properties

Total soluble solids (TSSs), pH values, total titratable acidity (TTA), and the salinity of traditionally fermented Thai vegetable products are shown in Table 3. The TSSs as soluble sugars consisted of monosaccharides, disaccharides, and polysaccharides along with minerals, acids, and vitamins [34] with results ranging from 2.92 to 21.90 °Brix. Pickled ginger had the highest TSS value of 21.90 °Brix, significantly different from the other samples, and pickled shallot recorded a TSS value of 12.61 °Brix. Thus, both pickled ginger and pickled shallot contained high amounts of soluble sugars. The other fermented samples had TSS measurements of less than 5 °Brix and were not significantly different.

The pH values ranged from 3.22 to 4.28, and the total titratable acidity (TTA), expressed as lactic acid, ranged from 0.37% to 1.35%. The pH and acidity levels play a crucial role in the process of fermentation and have a direct influence on microbial growth and enzyme activity. Lower pH or higher acidity levels create a more favorable environment for the growth of microorganisms, leading to accelerated fermentation and increased enzyme activity [35,36]. The pickled shallot and pickled ginger samples had the highest total titratable acidity (TTA).

Most fermented samples had pH values below 4, except for fermented tea leaves and pickled bud rieng. The primary fermentation technique involves layering food with salt to draw out the moisture and brine pickling which entails submerging food in a saltwater solution. A significant water loss results from the high salinity found in fermented vegetables [37]. The highest salinity level was recorded in pickled ginger (20.91%) followed by shallot (13.85%). The salinity percentages in the other fermented samples were 4% lower with no significant difference.

### 3.2. Nutritional Properties

Table 4 presents the biochemical compositions of the 10 traditionally fermented Thai vegetable products. Fermented vegetables collected from different regions in Thailand exhibited varying nutritional properties. The fermented vegetables had carbohydrate contents ranging from 10.73% to 75.25% dry weight. Pickled ginger and pickled shallot showed the highest carbohydrate composition with no significant difference. Lipid contents ranged from 1.03% to 33.90% dry weight, with pickled stink beans displaying the highest, followed by pickled bud rieng. Pickled stink beans exhibited high lipid and protein compositions. Protein contents ranged from 0.29% to 7.58% dry weight, with pickled stink beans and pickled bud rieng showing the highest protein content with no significant difference. Ash content of the fermented vegetables ranged from 8.86% to 41.64% dry weight. This diversity in nutritional properties highlights the richness and variety of traditionally fermented Thai vegetable products.

### 3.3. TPC and Antioxidant Activities

Vegetables provide important natural phenolic antioxidants that are recognized for their beneficial health properties. This research on traditionally fermented Thai vegetable products revealed insightful data on their total phenolic contents and antioxidant properties, as detailed in Table 5. The total phenolic content (TPC) ranged from 199.54 to 871.50 µg GAE/g dry weight, with the highest TPC observed in fermented tea leaves. The results indicate the significant presence of phenolic compounds in these fermented vegetables with known potential health benefits. The antioxidant properties were evaluated using the DPPH and ABTS assays. The DPPH assay demonstrated scavenging inhibition ranging from 51.34% to 83.77%, indicating the ability of the fermented products to act as DPPH free radical scavengers.

By contrast, the ABTS assay results ranged from 29.95% to 94.57%, indicating the diverse antioxidant capacities of the fermented vegetable products. Pickled bamboo shoots, mustard greens, and fermented tea leaves exhibited high antioxidant properties. The results suggest the potential health-promoting qualities of these fermented vegetable products, with statistically insignificant differences in antioxidant properties measured by the DPPH and ABTS assays. These fermented vegetable products possessed balanced antioxidant properties, making them potentially valuable dietary additions.

### 3.4. Comparison of Overall Profiles

The chemical profiles of the vegetables evaluated by univariate analysis and multivariate statistical approaches were significantly different. HCA and PLS-DA were applied to explore the similarities and distinctions in chemical profiles among the different product types. Pickled shallot and pickled ginger and another two samples as pickled stink beans and pickled bud rieng exhibited separate chemical profile patterns compared to the other products. PLS-DA-derived VIP scores emphasized the significance of lipid, TTA, protein, fiber, salinity, and TTS components in defining the distinctive characteristics of fermented vegetable products. The Pearson’s correlation coefficient matrix and correlogram provided insights into the relationships between these components and the chemical parameters determined in this study. 

**Figure 3:**
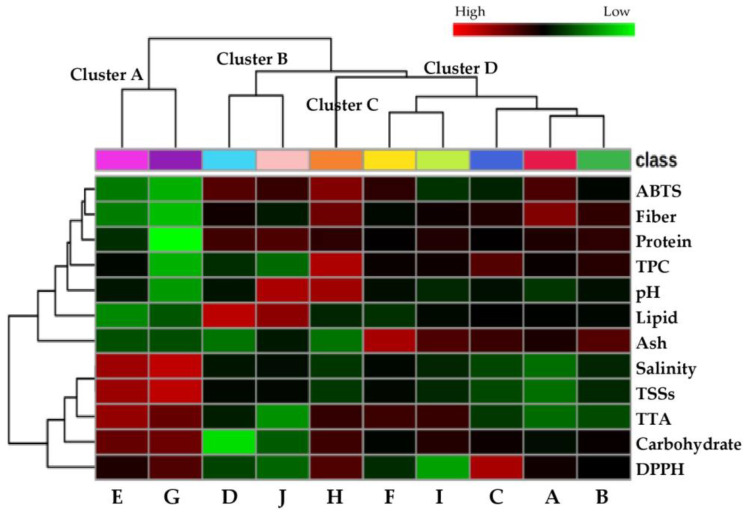
Heatmap visualization and hierarchical clustering of the chemical profiles of pickled pak-kum (A; 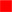), pickled pak-sian (B; 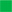), pickled bamboo shoots (C; 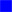), pickled stink beans (D; 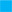), pickled shallot (E; 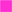), pickled mustard greens (F; 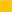), pickled ginger (G; 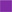), fermented tea leaves (H; 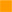), pickled scallions (I; 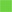), and pickled bud rieng (J; 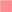) samples. The dendrogram represents sample clusters based on Pearson’s correlation coefficient with average linkage. Each square in the heatmap expresses normalized chemical abundance as the color range. The red color indicates a higher content of the corresponding chemical parameter. To better interpret the references to color in this figure, please see the statistical comparisons of the sample chemical properties in Table 3, Table 4 and Table 5.

A nonsupervised Pearson’s correlation-based hierarchical clustering analysis combined with a heatmap visualization was performed to evaluate the overall similarities among the chemical profiles of traditionally fermented Thai vegetable samples (Figure 3). The results demonstrate clear distinctive patterns for the chemical profiles of pickled shallot and pickled ginger (cluster A), pickled stink beans and pickled bud rieng (cluster B), and fermented tea leaves (cluster C) compared to the other types of fermented vegetables (cluster D). The chemical parameters were also classified into two main clusters based on their variations among the samples. Different colors in the heatmap indicate the relative abundance of chemical characteristics observed among the samples. Red indicates a higher abundance, and green indicates a lower abundance of the respective chemical features. Color shading in the heatmap revealed higher levels of salinity, TSSs, TTA, carbohydrate, and DPPH with lower levels of ABTS, fiber, protein, TPC, pH, lipid, and ash in the pickled shallot and pickled ginger samples (cluster A). Therefore, these two samples were easily distinguished from the other products.

**Figure 4:**
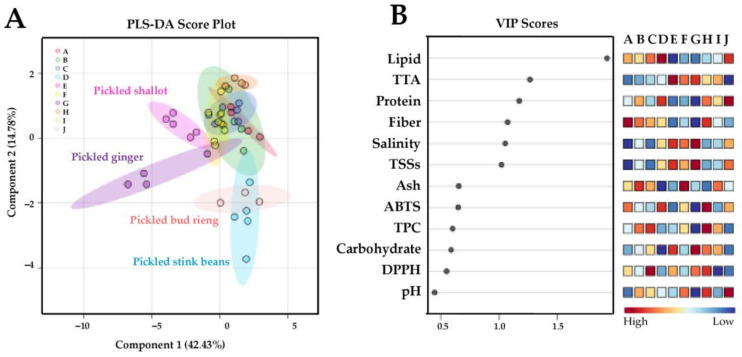
PLS-DA score plot (**A**) comparing the chemical profiles of pickled pak-kum (A; 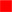), pickled pak-sian (B; 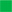), pickled bamboo shoots (C; 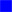), pickled stink beans (D; 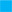), pickled shallot (E; 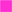), pickled mustard greens (F; 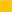), pickled ginger (G; 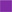), fermented tea leaves (H; 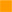), pickled scallions (I; 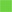), and pickled bud rieng (J; 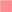) samples. Important features are organized in descending order of variable importance in projection (VIP) scores (**B**). Squares in the VIP score panel express normalized chemical abundance with respect to the color range. The red color indicates a higher content of the corresponding chemical parameter. To better interpret the references to color in this figure, please see the statistical comparisons of the sample chemical properties in Table 2, Table 3 and Table 4.

A supervised pattern recognition by PLS-DA was performed to discriminate the chemical profiles among different groups of fermented vegetables (Figure 4). An overall PLS-DA score plot was constructed using the first two components with a prediction accuracy of 68.88%, R^2^ = 0.467, and Q^2^ = 0.395 (Figure 3). The results demonstrate distinctive chemical profile patterns of the pickled shallot, pickled ginger, pickled stink bean, and pickled bud rieng samples. These four products were separated into two groups along component 1 (42.43%). The first group consisted of pickled shallot and pickled ginger which showed good discrimination. The second group consisted of overlapping pickled stink bean and pickled bud rieng samples. This result corresponds well with the HCA-derived dendrogram pattern mentioned above. PLS-DA-derived VIP scores with values greater than 1.0 (Figure 3) and *p* < 0.05 (Table 3 and Table 4) suggested that variations in lipid, TTA, protein, fiber, salinity, and TTS contents could be used to differentiate the fermented vegetable products in this study. 

### 3.5. Correlation Analysis among Different Chemical Parameters

**Figure 5:**
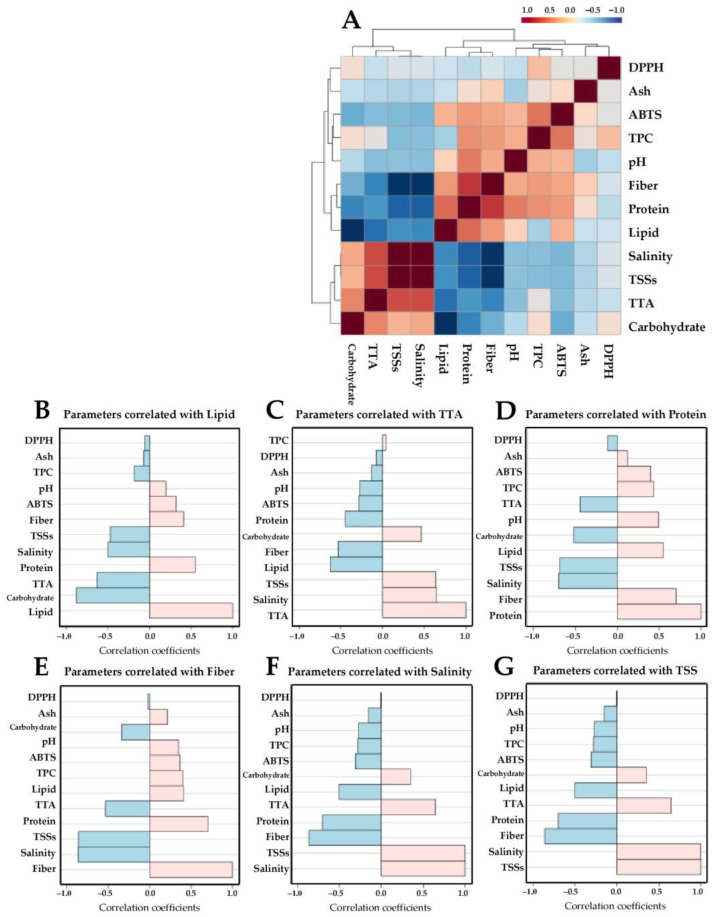
Correlogram of the 12 chemical parameters using Pearson’s correlation-based hierarchical clustering (**A**). Correlation coefficients are indicated in each colored cell on the map. The red and blue colors in the scale code at the top indicate positive and negative correlations, respectively. Correlation coefficient plots of the 12 chemical parameters (**B**–**G**) are represented as horizontal bars, with light pink indicating positive correlations and light blue indicating negative correlations to lipid (**B**), TTA (**C**), protein (**D**), fiber (**E**), salinity (**F**), and TSSs (**G**).

The 10 traditionally fermented Thai vegetable products were analyzed. A Pearson’s correlation coefficient matrix was constructed for the 12 chemical parameters and depicted as a correlogram (Figure 4A). The results reveal two main clusters with positively correlated (red shading) and negatively correlated (blue shading) parameters. Chemical features with a PLS-DA-derived VIP score greater than 1.0, i.e., lipid, TTA, protein, fiber, salinity, and TTSs were selected to demonstrate their correlation coefficients with other parameters. A strong association between parameters was considered when Pearson’s correlation coefficient was ≥0.5. Lipid content was positively correlated with protein and negatively correlated with TTA and carbohydrate levels (Figure 4B). TTA was positively correlated with TSSs and salinity and negatively correlated with fiber and lipid contents (Figure 5C). Protein level was positively correlated with fiber and lipid and negatively correlated with salinity and TSSs (Figure 5D). Fiber content was positively correlated with protein and negatively correlated with salinity, TSSs, and TTA (Figure 5E). Salinity was positively correlated with TSSs and TTA and negatively correlated with fiber and protein levels (Figure 5F). TSSs were positively correlated with salinity and TTA and negatively correlated with fiber and protein contents (Figure 5G). 

## 4. Discussion

Consuming vegetables is highly recommended due to their numerous nutritional benefits. Fermented vegetables play a significant role in the global food supply [38]. Fermentation, one of the oldest food preservation techniques, effectively extends the shelf life of vegetables by inducing changes in the variety and composition of bioactive compounds [39]. The fermentation of vegetables creates a wide array of healthy and delicious food options, thereby improving the overall nutritional content of the human diet by eliminating substances that hinder nutrient absorption [40]. This research subjected 10 traditionally fermented Thai vegetables to a thorough analysis. The physicochemical, nutritional, and antioxidant properties of the fermented vegetables were assessed for their potential health benefits and significant roles in culinary practices. Our findings reveal that traditionally fermented vegetable products commonly found in the northern region included pickled pak-kum and fermented tea leaves. By contrast, pickled scallions were prevalent in both the northern and northeastern regions. Pickled pak-sian and pickled ginger were found in the northeastern and central regions, respectively, while pickled stink beans and pickled bud rieng were only found in the southern region. Pickled bamboo shoots, pickled shallot, and pickled mustard greens were found in various forms across all regions of the country. Traditional fermentation refers to the natural and spontaneous process that occurs with the growth of microorganisms and metabolites as their byproducts [41]. The traditional process of fermenting vegetables leads to the creation of a diverse range of products as the specific types of locally available vegetables. 

Traditional vegetables show significant diversity across different global regions, each with its unique selection. Thailand has a distinctive assortment of traditional vegetables desired by consumers. These vegetables can be classified into two main groups based on their fermentation processes as naturally and artificially inoculated [42]. Our findings show that the natural fermentation process of different types of vegetables resulted in varying levels of TSSs, pH, total titratable acidity (TTA), and salinity. These variations were attributed to the distinctive physicochemical properties of the different vegetable materials used in fermentation. Traditional vegetables naturally fermented by microorganisms and lactic acid bacteria undergo physicochemical changes involving the conversion of sugars and nutrients in the vegetable material into lactic acid under anaerobic conditions [9,43]. The TSS value refers to the carbohydrate-soluble sugar. Our results show TSS values lower than 6 in many traditionally fermented vegetables. The TSS value decreased after the natural fermentation process of mixed fruit products and then stabilized, which was similar to the results of our study [34]. Our results show that pickled ginger and pickled shallot had high TSSs, TTA, and salinity. The notable increase in TSS content within the fermented products was attributed to the water stress or salinity encountered during the initial stages of the fermentation process, which were integral components of the fermentation process. Environmental conditions such as limited water availability or high salinity impact the fermentation process, leading to a higher TSS content in the end products [44]. The TSS contents of fermented green asparagus roots were 11.2 to 19.8 °Brix, similar to our results from pickled ginger [45].

The physicochemical properties of traditionally fermented vegetable products are intricately linked to their nutritional characteristics. An observed correlation indicated that elevated TSS content in these products directly influenced higher carbohydrate levels. Carbohydrates occur in plants as monosaccharides, oligosaccharides, and polysaccharides and can degraded to monosaccharides or lactic acid is the main product of lactic acid bacteria [46]. These monosaccharides are measured by the total soluble solid content in fermented foods [34,47]. This connection underscored the substantial impact of fermentation on the composition of traditionally fermented pickled ginger and pickled shallot vegetable products. Pickled stink beans and bud rieng had a high protein content, comparable to stink bean flour with higher protein levels and a lower carbohydrate content [48,49]. Both pickled stink beans and bud rieng are used as traditional vegetable materials and have a high lipid content. A report on fermented plant-based milk from beans found unsaturated fatty acids with linoleic acid, α-linolenic acid, and oleic acid [50]. A high fiber content was obtained from the leaves of traditional vegetables, which were used as raw material in the fermentation process. These fermented vegetables are natural sources of fiber, and enhance the nutritional value of products by promoting digestive health and overall well-being. The plant fibers play a key role in gut fermentation [51]. Dietary fiber is fermented by human gut microbiota and leads to the production of beneficial microbial metabolites, especially short-chain fatty acids. This process significantly promotes gut health and overall well-being [52]. 

The fermentation process impacts the biological characteristics of plant-based foods [53]. Antioxidants prevent the development of chronic diseases such as cancer, cardiovascular diseases (hypertension), and the pathogenesis of immunodeficiency viruses. Antioxidants neutralize harmful molecules in the body known as free radicals, which damage cells and lead to the development of diseases. Antioxidants play a crucial role in maintaining overall health and reducing the risk of serious conditions by reducing oxidative stress and inflammation [54,55]. Our results show varying biological property values of TPC, DPPH, and ABTS depending on the types of vegetables and the microorganism strains during natural fermentation [56]. Previous reports showed that the fermentation process had a beneficial impact on the characteristics and arrangement of phenolic compounds and antioxidant properties [57,58]. Fermentation resulted in higher levels of total phenolic content, total flavonoids, total FRAP, and ORAC values, ultimately leading to increased antioxidant activity [59]. 

The production processes for the fermented vegetables varied. The fundamental processing stages involved the initial treatment of the raw materials as brining, seasoning, and natural or starter culture-assisted fermentation, which lasted from several days to several months [11,60]. Predominant fermentation methods included dry salting and brine pickling. The quality of fermented vegetables is influenced by the metabolic activities of the microbial population, comprising lactic acid bacteria and yeast, during the fermentation process. This microbial community and the enzymatic activity, seasonal diversity, and fermentation conditions collectively impact the quality of the final product [37]. Moreover, lactic acid bacteria (LAB) such as *Lactococcus*, specifically *Lactiplantibacillus plantarum/pentosus*, *Levilactobacillus brevis*, *Leuconostoc mesenteroides*, *Pediococcus pentosaceus*, *Limosilactobacillus fermentum*, *Weissella*, *Leuconostoc*, *Pediococcus*, and *Lactococcus lactis* are the predominant species in the microbial community of fermented vegetables [61,62]. Lactic acid bacteria are essential for producing high-quality fermented vegetables. They can be classified into distinct groups based on their environment as homofermenters, heterofermenters, and facultative fermenters [46]. Lactic fermentation is the key process during vegetable fermentation, and it can be further categorized into homotypic and heterotypic fermentation pathways through the phosphoenolpyruvate-dependent sugar phosphotransferase system which is related to several enzymes [26,46,63]. Fermented vegetables offer various health benefits such as fighting off bacteria, easing constipation, preventing cancer, managing chronic diseases, relieving irritable bowel syndrome, and boosting the immune system [37]. Consequently, traditionally fermented Thai vegetables are an excellent option for probiotic products and functional foods. Our results advance food research by enhancing the quality and preserving the authenticity of traditionally fermented Thai vegetables, leading to the beneficial future development of these food products. The consumption of fermented vegetable products is deeply rooted in human nutrition, with significance in diverse cultural customs. Vegetables are highly perishable due to their elevated water content and are particularly susceptible to spoilage, especially in tropical and subtropical regions. Lactic acid fermentation has emerged as a pivotal method for prolonging the shelf life of fruits and vegetables, concurrently enriching their nutritional profile, flavor, and safety by mitigating toxicity. Lactic acid bacteria in fermented vegetables are a source of probiotics. Traditionally fermented vegetables serve as dietary complements and offer notable health benefits [11]. This study demonstrates the effectiveness of applying multidimensional statistical approaches to characterize the chemical profiles of fermented vegetable products. Understanding these chemical signatures facilitates quality control and authentication, providing valuable insights for product development and future optimization.

## 5. Conclusions

Our research thoroughly analyzed ten types of traditionally fermented commercial Thai vegetables to assess their physicochemical properties, encompassing nutritional values and antioxidant capacities. The results highlight the substantial impact of different vegetable materials from traditional fermentation on the diverse product attributes. Our results provide further evidence of the significant health benefits associated with microorganisms, particularly probiotics, in fermented foods. Furthermore, our findings confirm the elevated levels of total soluble solids, titratable acidity, and salinity in pickled shallot and ginger, marking the pronounced influence of the natural fermentation process and the choice of ingredients. The study also conclusively identifies variations in nutritional composition, total phenolic contents, and antioxidant activities among the different types of vegetables. Traditionally fermented Thai vegetables are undergoing further studies using the metabolomic approach.

## Figures and Tables

**Table 1:** Ten traditionally fermented Thai vegetables and local food collected across Thailand.

Traditionally Fermented Thai Vegetables (Local Name)	*N*	Scientific Name	Province	Region
Pickled pak-kum	*N* = 4	*Crateva adansonii*	Chiang Rai	Northern
(Pak-Kum Dong)			Tak	Northern
			Chiang Rai	Northern
			Trang	Southern
Pickled pak-sian	*N* = 5	*Cleome gynandra*	Nakhon Si Thammarat	Southern
(Pak-Sian Dong)			Amnat Charoen	Northeastern
			Ubon Ratchathani	Northeastern
			Ubon Ratchathani	Northeastern
			Trang	Southern
Pickled bamboo shoots (Nor-Mai Dong)	*N* = 5	*Bambusa vulgaris*	Lampang	Northern
			Prachuap Khiri Khan	Western
			Bangkok	Central
			Trang	Southern
			Chiang Mai	Northern
Pickled stink beans	*N* = 5	*Parkia speciosa*	Pattani	Southern
(Sator Dong)			Nakhon Si Thammarat	Southern
			Nakhon Si Thammarat	Southern
			Surat Thani	Southern
			Songkhla	Southern
Pickled shallot	*N* = 5	*Allium oschaninii*	Prachuap Khiri Khan	Western
(Homdang Dong)			Bangkok	Central
			Surat Thani	Southern
			Si Sa Ket	Northeastern
			Saraburi	Central
Pickled mustard greens (Pak-Kard Dong)	*N* = 7	*Brassica juncea*	Nakhon Pathom	Central
			Lampang	Northern
			Bangkok	Central
			Ratchaburi	Western
			Kanchanaburi	Western
			Chon Buri	Eastern
			Chiang Mai	Northern
Pickled ginger	*N* = 4	*Zingiber officinale*	Nakhon Pathom	Central
(King Dong)			Bangkok	Central
			Bangkok	Central
			Bangkok	Central
Fermented tea leaves	*N* = 4	*Camellia sinensis* var. *assamica*	Phrae	Northern
(Miang)			Tak	Northern
			Phayao	Northern
			Chiang Mai	Northern
Pickled scallions	*N* = 3	*Allium fistulosum*	Chiang Mai	Northern
(Ton-Hom Dong)			Amnat Charoen	Northeastern
			Nakhon Ratchasima	Northeastern
Pickled bud rieng	*N* = 3	*Parkia timoriana*	Phatthalung	Southern
(Nor-Rieng Dong)			Nakhon Si Thammarat	Southern
			Nakhon Si Thammarat	Southern

*N*: Number of traditionally fermented Thai vegetables collected.

**Table 2:** The overall fermentation process of ten traditionally fermented Thai vegetables.

Traditionally	Type	Part of Vegetables	Ingredients *	Fermentation Condition
Fermented Thai Vegetables				
(Local Name)				
Pickled pak-kum	A	Leaves	Salt	Room temperature, 3–7 days
(Pak-Kum Dong)			Rice-washed water	
			Cooked rice/Sticky rice	
Pickled pak-sian	B	Leaves	Salt	Room temperature, 3–7 days
(Pak-Sian Dong)			Rice-washed water	
Pickled bamboo shoots	C	Tuber	Salt	Room temperature, 3–5 days
(Nor-Mai Dong)			Rice-washed water	
Pickled stink beans	D	Seeds	Salt	Room temperature, 3–5 days
(Sator Dong)			Sugar	
			Water/Rice-washed water	
Pickled shallot	E	Tuber	Salt	Room temperature, 5–10 days
(Homdang Dong)			Sugar	
			Water/Rice-washed water	
			Vinegar	
Pickled mustard greens	F	Leaves	Salt	Room temperature, 3–10 days
(Pak-Kard Dong)			Rice-washed water	
Pickled ginger	G	Tuber	Salt	Room temperature, 5–7 days
(King Dong)			Sugar	
			Vinegar	
			Water	
Fermented tea leaves	H	Leaves	Salt	Room temperature, 3–4 months
(Miang)			Water	
			Vinegar	
Pickled scallions	I	Leaves	Salt	Room temperature, 5–7 days
(Ton-Hom Dong)			Rice-washed water	
			Cooked rice/Sticky rice	
Pickled bud rieng	J	Seeds	Germinated seeds	Room temperature, 2–7 days
(Nor-Rieng Dong)			Salt	
			Sugar	
			Water/Rice-washed water	

* The ingredients vary by producer and may or may not contain any types.

**Table 3:** Physicochemical properties of the ten traditionally fermented Thai vegetables.

Traditionally Fermented Thai Vegetables	TSSs (°Brix)	pH	TTA (%)	Salinity (%)
Pickled pak-kum	2.92 ± 0.60 ^c^	3.51 ± 0.33 ^b^	0.37 ± 0.11 ^d^	2.40 ± 0.49 ^c^
Pickled pak-sian	4.15 ± 0.56 ^c^	3.72 ± 0.53 ^b^	0.37 ± 0.05 ^d^	3.47 ± 0.45 ^c^
Pickled bamboo shoots	5.21 ± 1.40 ^c^	3.62 ± 0.28 ^b^	0.53 ± 0.12 ^d^	4.33 ± 1.18 ^c^
Pickled stink beans	5.41 ± 1.61 ^c^	3.61 ± 0.20 ^b^	0.56 ± 0.15 ^cd^	4.83 ± 0.78 ^c^
Pickled shallot	12.61 ± 3.72 ^b^	3.60 ± 0.20 ^b^	1.35 ± 0.29 ^a^	13.85 ± 3.19 ^b^
Pickled mustard greens	5.62 ± 1.14 ^c^	3.63 ± 0.21 ^b^	1.07 ± 0.30 ^ab^	4.70 ± 0.97 ^c^
Pickled ginger	21.90 ± 2.86 ^a^	3.22 ± 0.14 ^b^	1.35 ± 0.37 ^a^	20.91 ± 0.07 ^a^
Fermented tea leaves	4.44 ± 1.13 ^c^	4.20 ± 0.28 ^a^	0.85 ± 0.05 ^bc^	3.68 ± 0.96 ^c^
Pickled scallions	4.78 ± 1.31 ^c^	3.54 ± 0.14 ^b^	1.01 ± 0.06 ^b^	4.02 ± 1.09 ^c^
Pickled bud rieng	4.33 ± 0.72 ^c^	4.28 ± 0.80 ^a^	0.45 ± 0.03 ^d^	3.47 ± 0.58 ^c^

Data in the same column with different superscripts are significantly different (*p* < 0.05).

**Table 4:** Nutritional properties of the 10 traditionally fermented Thai vegetables (g/100 g dry weight).

Traditionally Fermented Thai Vegetables	Crude Lipid	Crude Protein	Crude Fiber	Ash	Carbohydrate
Pickled pak-kum	4.79 ± 0.86 ^cd^	4.77 ± 0.65 ^b^	24.44 ± 3.73 ^a^	20.48 ± 2.70 ^bc^	43.71 ± 4.81 ^bc^
Pickled pak-sian	5.90 ± 0.71 ^c^	5.65 ± 1.23 ^b^	16.53 ± 3.68 ^b^	27.23 ± 5.43 ^b^	41.37 ± 8.09 ^bc^
Pickled bamboo shoots	4.27 ± 1.27 ^cde^	3.37 ± 0.65 ^c^	15.23 ± 3.71 ^bc^	37.36 ± 8.23 ^a^	42.76 ± 12.69 ^bc^
Pickled stink beans	33.90 ± 1.29 ^a^	6.75 ± 0.24 ^a^	14.11 ± 3.10 ^bc^	9.96 ± 0.96 ^d^	10.73 ± 0.05 ^d^
Pickled shallot	0.62 ± 0.05 ^f^	2.38 ± 0.44 ^d^	6.26 ± 3.22 ^d^	13.44 ± 5.01 ^cd^	72.90 ± 5.84 ^a^
Pickled mustard greens	2.57 ± 0.44 ^ef^	3.76 ± 0.54 ^c^	12.20 ± 1.72 ^bc^	41.64 ± 6.03 ^a^	37.60 ± 5.14 ^c^
Pickled ginger	1.03 ± 0.10 ^f^	0.29 ± 0.13 ^e^	3.77 ± 0.43 ^d^	9.31 ± 3.02 ^d^	75.25 ± 2.35 ^a^
Fermented tea leaves	2.65 ± 0.28 ^def^	5.59 ± 0.81 ^b^	22.19 ± 4.29 ^d^	8.86 ± 4.16 ^d^	55.55 ± 6.19 ^b^
Pickled scallions	3.70 ± 0.30 ^de^	4.96 ± 0.11 ^b^	13.83 ± 2.09 ^b^	26.45 ± 5.95 ^b^	47.63 ± 3.59 ^bc^
Pickled bud rieng	21.96 ± 5.10 ^b^	7.58 ± 1.17 ^a^	11.48 ± 2.97 ^d^	11.57 ± 2.60 ^d^	22.32 ± 1.36 ^d^

Data in the same column with different superscripts are significantly different (*p* < 0.05).

**Table 5:** Total phenolic content and antioxidant properties of the 10 traditionally fermented Thai vegetables.

Traditionally Fermented Thai Vegetables	TPC (µg GAE/g)	DPPH (% Inhibition)	ABTS (% Decolorization)
Pickled pak-kum	434.19 ± 70.52 ^cd^	67.40 ± 9.67 ^bc^	77.43 ± 3.00 ^b^
Pickled pak-sian	504.66 ± 33.77 ^bc^	76.62 ± 1.35 ^abc^	72.96 ± 6.08 ^b^
Pickled bamboo shoots	599.81 ± 70.21 ^b^	81.41 ± 3.85 ^ab^	94.37 ± 0.11 ^a^
Pickled stink beans	347.71 ± 82.79 ^de^	51.34 ± 8.06 ^d^	79.74 ± 9.68 ^b^
Pickled shallot	496.94 ± 55.65 ^bc^	66.71 ± 6.55 ^c^	49.68 ± 5.88 ^c^
Pickled mustard greens	490.42 ± 64.55 ^bc^	83.77 ± 2.86 ^a^	94.26 ± 0.12 ^a^
Pickled ginger	199.54 ± 50.68 ^f^	72.30 ± 3.31 ^abc^	29.95 ± 8.12 ^d^
Fermented tea leaves	871.50 ± 80.25 ^a^	71.99 ± 8.96 ^abc^	94.57 ± 0.26 ^a^
Pickled scallions	449.19 ± 80.60 ^cd^	78.92 ± 4.66 ^abc^	38.06 ± 0.92 ^d^
Pickled bud rieng	264.59 ± 40.20 ^ef^	65.34 ± 9.16 ^c^	70.31 ± 6.58 ^b^

Data in the same column with different superscripts are significantly different (*p* < 0.05).

## Data Availability

All original contributions are included in this article. Further inquiries can be directed to the corresponding author.
